# Editorial: Endothelial dysfunction in endocrine disorders

**DOI:** 10.3389/fendo.2023.1321072

**Published:** 2023-10-31

**Authors:** Miroslav Radenković, Zhice Xu

**Affiliations:** ^1^ Wuxi Women and Child Care Hospital, Perinatal Medicine Lab, Wuxi, China; ^2^ Department of Pharmacology, Clinical Pharmacology and Toxicology, Faculty of Medicine – University of Belgrade, Belgrade, Serbia; ^3^ Institute for Fetology, First Hospital of Soochow University, Jiangsu, China

**Keywords:** endothelium, endothelial dysfunction, endocrine disorders, preeclampsia, thyrotoxicosis

Endothelial dysfunction is a pathological condition principally characterized by impaired endothelium–dependent transduction mechanisms related to vascular relaxation and other important physiological functions, as an outcome of decreased release of endothelium-derived relaxing factors, augmented oxidative stress, increased inflammation and predominance of vascular action produced by endothelium-derived contracting factors. Current data strongly suggest that endocrine pathological disorders comprise notable pathological changes associated with endothelial dysfunction. Therefore, the aim of this Research Topic was to present some of the latest experimental and clinical findings related to this scientific topic.

Tang et al. comprehensively investigated the physiological features of placental vessels related to the NO/sGC role, and provided an original results and important information on preeclampsia onset. Endothelium-dependent vasodilators, including nitric oxide (NO), mediated very limited vascular dilation in the placenta, significantly different from that of non-placental vascular endothelium. This kind of finding also is a serious and important challenge to the classic theory “Placental endothelial dysfunction leading to the development of preeclampsia”. However, exogenous NO provided by sodium nitroprusside can produce significant dilation at basal levels in placental micro-vessels, also very different from non-placental blood vessels. Those results demonstrated that non-endothelial (exogenous) NO played important roles via sGC in regulations of placental vascular baseline tone. The lower NO production and decreased NO/sGC pathway may be one of reasons for the onset of preeclampsia. The results contribute to understanding specific feature of umbilical-placental blood vessels and provide pathophysiological information on preeclampsia at levels of placental vessels.

Phowira et al. provided an original research aimed to evaluate whether endothelial function, endothelial progenitor cells (cEPC) and circulating angiogenic cells (CAC) counts were reduced in subclinical thyrotoxicosis (SCT) and to study the *in vitro* effect of triiodothyronine (T3) on proangiogenic cell (PAC) function from young healthy controls. This was substantiated by the fact that the treatment of SCT is still uncertain despite being associated with increased cardiovascular risk (CVR) and mortality, where cEPCs have been suggested to play an important role in endothelial regeneration. By using well chosen methodological approach it was reported for the first time that SCT was indeed associated with a lower cEPC count and reduced flow-mediated dilation (FMD), thus confirming that patients with SCT display poor vascular health *in vivo* and *in vitro*, which is consistent with increased CVR.

Cong et al. were aimed to investigate the effects of acute angle closure crisis (AACC) on the corneal endothelial cells in patients with type 2 diabetes mellitus (DM) to identify the factors that cause corneal endothelial cell injury. AACC is an ophthalmic emergency with a high incidence rate in middle-aged and elderly persons, which is still under investigation. At the same time, the main manifestations of diabetic keratopathy include decreased corneal nerve sensitivity, corneal epithelial injury, and abnormal corneal endothelial cell function. In a group of 154 participants it was found that DM may impact the functional status of corneal endothelial cells, where AACC can worsen the corneal endothelium damage in patients with DM. Blood glucose levels and the duration of intraocular hypertension were closely related to the severity of corneal endothelial injury, which requires continuous education of all parties involved for the effective prevention and adequate control.

Zhang et al. underlined that limited studies have characterized how maternal exercise influences endothelial function of hypertensive offspring, therefore providing a piece of detailed experimental evidence clearly arguing that maternal exercise represses NADPH oxidase-4 (Nox4) via sirtuin 1 (SIRT1) to prevent vascular oxidative stress and endothelial dysfunction in spontaneously hypertensive rats (SHR) offspring. The authors provided a wide-ranging methodological approach to fully examine the initial hypothesis with detailed insight into the epigenetic mechanism linking maternal exercise to fetal development and endothelial function, as well as long-term vascular health in hypertensive offspring. The clinical contribution of this paper lies in the evident notion that prenatal exercise should be accounted as a novel therapeutic approach to limit the inheritance of hypertension or other cardiovascular diseases to the next generation.

Wang et al. submitted a comprehensive review regarding the significance of endothelial-to-mesenchymal transition in key diabetic complications, the specific mechanisms at play, and potential mechanisms to prevent endothelial-to-mesenchymal transition. This was supported by up-to-date knowledge that endothelial-to-mesenchymal transition represents a tightly regulated process resulting in endothelial cells pathological change and developing mesenchymal traits. Even though this phenomenon has been found to take place within most of the major complications of diabetes, it has not been a major focus of study or a common target in the treatment or prevention of diabetic complications. The authors provided a valuable contribution in analyzing the myriad pathways involved in mediate endothelial-to-mesenchymal transition, as well as the epigenetic regulations that maintain this process.

Ding et al. contributed with the systematic review and the meta-analysis on vascular endothelial growth factor levels in diabetic peripheral neuropathy. It was of interest to investigate the relationship among cycling levels of vascular endothelial growth factors (VEGFs) and diabetic peripheral neuropathy (DPN). Since so far results were inconsistent, the presented analysis provided an additional value to this critical scientific problem with important clinical repercussions. The authors underlined that compared with healthy people and diabetic patients without DPN, VEGF content in the peripheral blood of DPN patients was increased, but existing evidence does not support the link in-between VEGF levels and the risk of DPN. This implies that VEGF may actually play a role in the pathogenesis and restoring of DPN. Given the several very well underlined limitations of this study, careful consideration and additional investigations are still expected to notably clarify any uncertainties in respect to DPN and VEGF.

## Author contributions

MR: Writing – original draft, Writing – review & editing. ZX: Writing – original draft, Writing – review & editing.

